# Decomposing Kenyan socio-economic inequalities in skilled birth attendance and measles immunization

**DOI:** 10.1186/1475-9276-12-3

**Published:** 2013-01-07

**Authors:** Carine Van Malderen, Irene Ogali, Anne Khasakhala, Stephen N Muchiri, Corey Sparks, Herman Van Oyen, Niko Speybroeck

**Affiliations:** 1 Institute of Health and Society (IRSS), Université catholique de Louvain, Brussels, Belgium; 2 Veterinary Research Centre, Kenya Agricultural Research Institute, Kikuyu, Kenya; 3 Population Studies Research Institute (PSRI), University of Nairobi, Nairobi, Kenya; 4 Futures Group, Nairobi, Kenya; 5 Department of Demography, University of Texas, San Antonio, USA; 6 Unit of Public Health and Surveillance, Scientific Institute of Public Health, Brussels, Belgium

**Keywords:** Kenya, Skilled birth attendance, Measles immunization, Socio-economic inequalities, Concentration index, Decomposition

## Abstract

**Introduction:**

Skilled birth attendance (SBA) and measles immunization reflect two aspects of a health system. In Kenya, their national coverage gaps are substantial but could be largely improved if the total population had the same coverage as the wealthiest quintile. A decomposition analysis allows identifying the factors that influence these wealth-related inequalities in order to develop appropriate policy responses. The main objective of the study was to decompose wealth-related inequalities in SBA and measles immunization into their contributing factors.

**Methods:**

Data from the Kenyan Demographic and Health Survey 2008/09 were used. The study investigated the effects of socio-economic determinants on [1] coverage and [2] wealth-related inequalities of SBA utilization and measles immunization. Techniques used were multivariate logistic regression and decomposition of the concentration index (C).

**Results:**

SBA utilization and measles immunization coverage differed according to household wealth, parent’s education, skilled antenatal care visits, birth order and father’s occupation. SBA utilization further differed across provinces and ethnic groups. The overall C for SBA was 0.14 and was mostly explained by wealth (40%), parent’s education (28%), antenatal care (9%), and province (6%). The overall C for measles immunization was 0.08 and was mostly explained by wealth (60%), birth order (33%), and parent’s education (28%). Rural residence (−19%) reduced this inequality.

**Conclusion:**

Both health care indicators require a broad strengthening of health systems with a special focus on disadvantaged sub-groups.

## Introduction

Skilled birth attendance (SBA) and measles immunization reflect two aspects of health systems. While the immunization of children requires an elaborate health delivery system and catch up campaigns (e.g., community immunization days), increasing levels of SBA can be more complex (requiring e.g., a minimum and continuous level of human resources for health), demanding broader efforts of the health system in place.

Both aforementioned aspects are of course important. In the African region, appropriate health care including SBA could greatly reduce maternal deaths and disabilities [1]. However, in many African countries, more than half of all births take place without the assistance of skilled health workers [2]. Routine measles vaccination coverage achieved >80% coverage in 2008, but a population immunity needs a coverage of >93–95% in all districts to prevent measles epidemics [3]. The WHO targets: >90% by routine at national level, >80% in all districts, and >95% by supplementary immunization activities (SIAs) in all districts [4] are not yet achieved.

Whereas efforts are being made to increase health care coverage in sub-Saharan countries, reaching the marginalized sub-populations may be the real future challenge. Indeed, socio-economic inequalities were observed in several health care indicators [5,6]. The question of whether to choose between a whole-population approach or more targeted interventions towards marginalized groups was raised [6]. Addressing these socio-economic health inequalities can be important not only from the social justice point of view but also from a general public health perspective as it may be the most efficient way to improve health care levels. This is particularly true for measles immunization: not reaching specific groups brings about sub-optimal coverage thereby maintaining the risk of epidemics, a risk for the whole population [7,8]. SBA and measles immunization are two indicators selected by the Countdown to 2015 Equity Analysis Group according to a number of criteria such as relevance to health system strengths [5]. The two indicators are as such also representing relevant indicators to monitor health care inequalities.

In Kenya, the national coverage gap (i.e., the increase in coverage required in order to achieve universal coverage) was 58% for SBA – among the highest compared to other African countries – and 27% for measles immunization (2008) [6]. The population attributable risk (PAR), i.e. the improvement possible if the total population has the same coverage as the wealthiest quintile, was 34% for SBA and 15% for measles immunization [6]. These figures indicate that the Kenyan population might benefit from a more targeted approach. The evidence on socio-economic inequalities in health and health care has led to renewed interest to understand the factors that influence these inequalities in order to develop appropriate policy responses [9-12]. Once inequalities have been observed, a logical step towards guiding interventions aimed at reducing inequalities is indeed to understand the observed differences, e.g., why do poor women have low access to health care programmes? Use of specific analytical tools, such as a decomposition analysis [13], is then needed in order to analyse the determinants of these inequalities. Such tools allow understanding how a determinant affects inequality: through its more unequal distribution across the population (e.g. illiteracy is more prevalent among poor mother) or through its greater association with the health outcome (e.g. mother’s illiteracy is associated with infant mortality) [13,14].

The main objective of this study was to decompose wealth-related inequalities in SBA and measles immunization into their contributing factors, the goal being to identify targets to lower these inequalities. The use of both health service indicators aims at entangling two different health service characteristics, namely SBA linked to the need of long-range investment programs requiring trained health professionals/facilities and measles immunization, which can be more easily addressed through community campaigns.

## Methods

### Study site and population

Kenya, located in the eastern part of Africa, is divided in 8 provinces. There are various ethnic groups and two main religions: Christianity and Islam. The economy is predominantly agricultural with a strong industrial base [15]. In 2008, the Gross National Income per capita (PPP) was $1560 [16] and the Gini index was 42.5 [17]. Forty% of the labour force was unemployed [17]. In 2009, the population was estimated at 39 million [16,18], 77% living in rural areas [16]. Adult literacy rate was 87% [16]. The number of women aged 15–49 years old was 9.6 million and the number of live births was 1.5 million. Fertility rate was estimated at 4.6 births per woman [19]. In 2008/09, 47% of women 20–24 gave birth before age 20 [15].

### Data

Data from the Demographic and Health Survey (DHS) conducted in Kenya in 2008/09 were used for the study. Data from preceding Kenyan DHS: 1993, 1998 and 2003 were used for a trends analysis. Data collection and processing are described in [15,20-22]. In brief, a two-stage sampling design stratified on region and place of residence (urban/rural) was used. Information on SBA (self-reported) was available for 4249, 2403, 5929 and 6059 children under 5 in 1993, 1998, 2003 and 2008/09 respectively. Information on measles immunization (recorded on the vaccination card or self-reported if not recorded) was available for 806, 780, 1096 and 1116 children aged 12–23 months in 1993, 1998, 2003 and 2008/09, respectively. The recall period was 5 years for both indicators but the measles analysis was restricted to children aged 12–23 months because this is the youngest cohort of children who have reached the age by which they should be fully vaccinated (<12 months) [15].

### Data analysis

Data were transferred to RGui (R version 2.14.2., The R foundation for Statistical Computing) for analysis. Both health care indicators were binary variables. SBA takes a value of 1 if the delivery has been attended by skilled health personnel (doctor, nurse or midwife). Measles immunization takes a value of 1 if the child aged >12-23 months has been immunized against measles. The independent variables used in the analysis were: antenatal care attendance, sex of the child, child’s age, birth order, mother’s age at birth, type of residence (urban/rural), province, ethnic group, religion, marital status, parents’ level of education, parents’ occupation, insurance coverage and household wealth. The wealth index, computed by DHS, comprises household assets (type of flooring, water supply, sanitation facilities, electricity, persons per sleeping room, ownership of agricultural land, domestic servant, and other assets).

The concentration index (C) [23] was used as a measure of socioeconomic inequality. The method is described in details elsewhere [11-13]. Briefly, a relative concentration curve plots on the x-axis the cumulative percentage of children ranked by household wealth and on the y-axis the cumulative percentage of the variable of interest (SBA, measles immunization or a determinant). The relative C is defined as twice the area between the concentration curve and the diagonal (line of equality). If the curve is below the diagonal, C is positive and the variable of interest is more prevalent among the wealthier households. Given that the bounds of the C of a binary health indicator depend on the mean of this indicator, a normalized C (C*) proposed by Erreygers was computed when comparing time periods or geographical areas [24].

In order to satisfy the linearity assumption of the decomposition method used [25], inequality in *predicted* SBA (ln odds SBA) and measles immunization was computed and decomposed into its contributing determinants [14]. For this, a multivariate logistic regression model was used to estimate the regression coefficients of each determinant on the two indicators, and compute the resulting predicted health care outcome. The C of the predicted health outcome can be expressed as:

$$C=\sum_{k}\left(\frac{\beta_{k}{\bar{x}}_{k}}{\mu }\right)C_{k}$$

the sum of contributions of the k determinants [13,25]. The contribution of each determinant is a function of its regression coefficient *β*_*k*_, its mean ${\bar{x}}_{k}$, the mean of the predicted health outcome*μ*, and its concentration index*C*_*k*_.

All analyses were weighted (weights provided with the DHS data) and adjusted for the cluster randomized sampling frame (with cluster as the primary sampling unit and household as the secondary sampling unit). The criterion for statistical significance used was α = 0.01.

## Results

### Comparison with other sub-Saharan African countries

**Figure 1:**
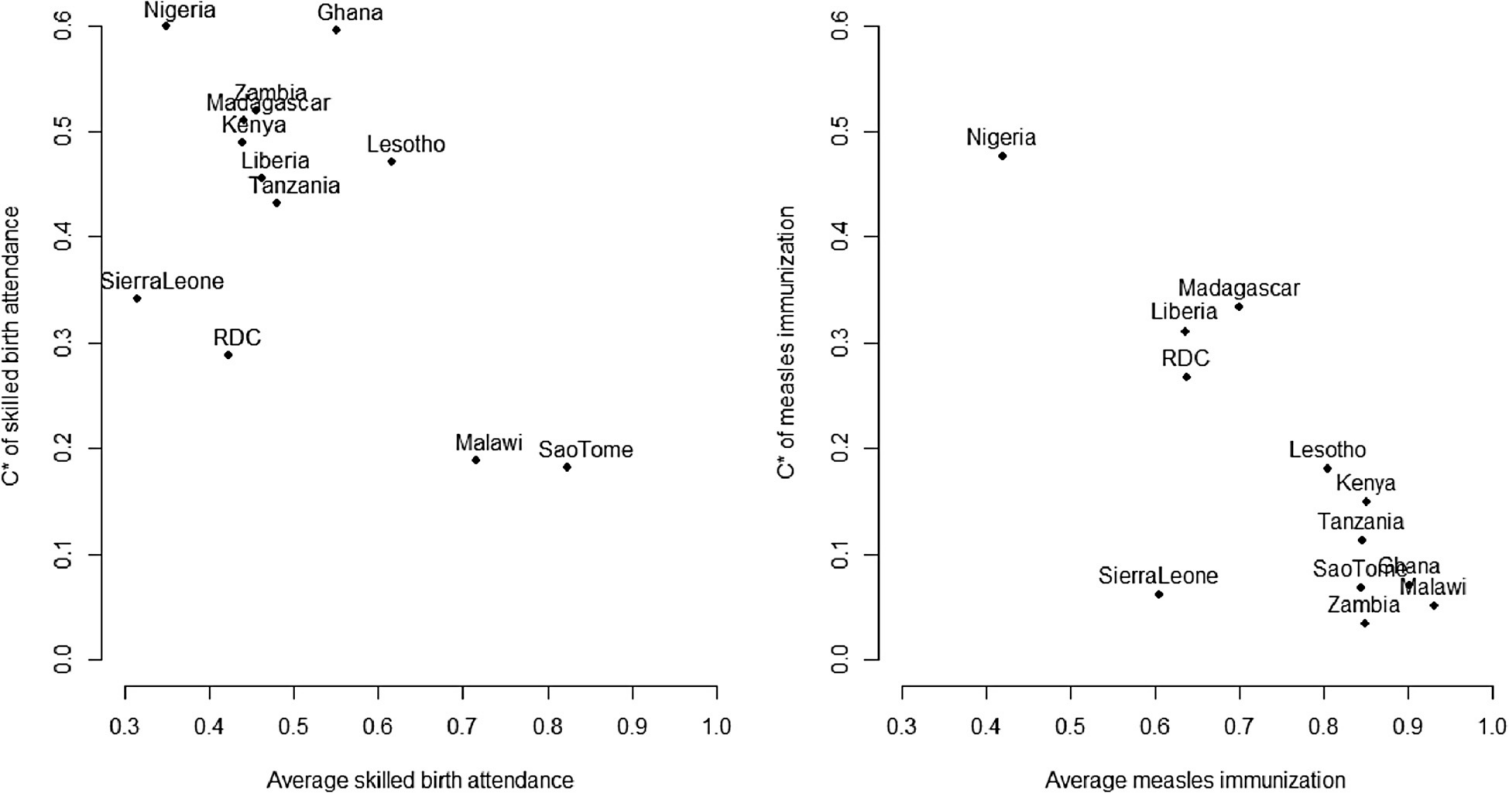
**Proportions of skilled birth attendance and measles immunization versus wealth-related inequality in these indicators, DHS 2007–2012.** * = Erreygers’ corrected concentration index [24].

Health care indicators means and concentration indices were computed in 11 other African countries with DHS data dating from 2007 to 2012 (Figure 1). Kenya was among countries with the lowest SBA (<50%) and the highest inequality in SBA (C* > 0.4). However, with respect to measles immunization coverage, Kenya ranked second to Malawi with the highest coverage (85%) and had a median position in measles coverage inequality.

### SBA, measles immunization and inequality by province

**Figure 2:**
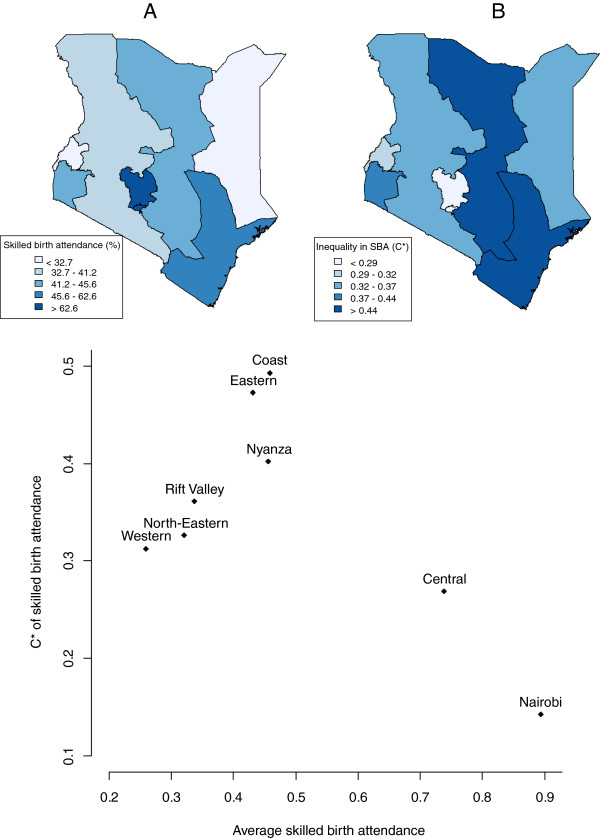
**Regional distributions of average (A) and inequality in (B) skilled birth attendance, Kenya, DHS 2008/09.** * = Erreygers’ corrected concentration index [24].

Nairobi and Central provinces had the highest percentages of SBA deliveries among women while North Eastern, Western and Rift Valley had the lowest (Table 1, Figure 2A). After controlling for the effect of the other variables, Rift Valley was still negatively associated with low uptake of SBA (Table 2). A relative normalized C was computed by province (Figures 2B). With the exception of the Central province and Nairobi, all provinces showed a low SBA coverage (<0.5) and a high inequality in SBA (C* > 0.3). Among this group of provinces, a positive association between coverage and inequality could be observed (R^2^: 0.79, p = 0.02). Coast and Eastern provinces had the highest inequality. Nairobi was the province with the highest coverage and the lowest inequality.

**Table 1:** Proportion of skilled birth attendance and measles immunization in total and by selected characteristics, Kenya, DHS 2008/09

	**Skilled birth attendance**		**Measles immunization**	
**Characteristics**	**%**	**(N)**	**%**	**(N)**
Total	43.9	(6059)	85.1	(1116)
Wealth quintile*^,§^				
1	20.4	(1768)	75.7	(313)
2	31.4	(1078)	80.8	(194)
3	42.0	(982)	85.5	(170)
4	52.9	(984)	90.0	(191)
5 (richest)	81.8	(1247)	93.9	(248)
Skilled antenatal visits*^,§^				
0	13.2	(428)	68.4	(115)
1 to 3	39.8	(1715)	83.4	(431)
4+	61.6	(1874)	88.6	(482)
Sex				
male	45.3	(3122)	84.2	(570)
female	43.3	(2937)	85.9	(546)
Child’s age				
12 to 17			81.4	(552)
18 to 23			88.5	(564)
Birth order*^,§^				
1	62.3	(1383)	91.8	(272)
2 to 3	46.6	(2279)	91.2	(404)
4 to 5	34.3	(1280)	78.1	(221)
6+	26.6	(1117)	71.8	(219)
Mother’s age at birth				
<20	45.3	(2950)	87.9	(544)
20+	42.6	(3109)	82.5	(572)
Type of residence*				
Rural	36.8	(4601)	83.4	(823)
Urban	75.2	(1458)	90.6	(293)
Province*				
Nairobi	89.3	(412)	87.6	(72)
Central	73.8	(496)	88.3	(81)
Coast	45.8	(880)	85.4	(155)
Eastern	43.1	(743)	88.7	(149)
North Eastern	32.1	(573)	80.0	(92)
Nyanza	45.5	(1109)	78.2	(204)
Rift Valley	33.7	(1058)	89.3	(210)
Western	25.9	(788)	77.7	(153)
Ethnic group*				
Kalenijn	37.2	(618)	88.1	(116)
Kamba	35.6	(400)	90.5	(73)
Kikuyu	75.3	(719)	91.1	(116)
Kisii	51.2	(309)	83.7	(58)
Luhya	28.9	(905)	84.5	(182)
Luo	45.6	(938)	75.1	(176)
Masai	26.6	(122)	59.6	(22)
Meru/Embu	68.8	(268)	92.7	(57)
Mijikenda	37.6	(596)	89.4	(107)
Taita	59.0	(68)	88.0	(12)
Other	29.0	(1114)	80.8	(197)
Religion				
Protestant	45.2	(3534)	84.7	(661)
Catholic	44.9	(1064)	87.3	(200)
Muslim	41.7	(1198)	85.2	(211)
Other	22.4	(254)	81.6	(43)
Marital status				
Married	43.8	(4822)	84.6	(892)
Other	44.3	(1237)	87.0	(224)
Mother’s education*^,§^				
Higher	88.3	(325)	94.3	(56)
Secondary	76.7	(549)	95.8	(105)
Secondary incomplete	59.5	(474)	85.7	(80)
Primary	48.9	(1514)	87.0	(296)
Primary incomplete	28.6	(1910)	80.1	(371)
No education	19.3	(1287)	79.1	(208)
Father’s education*^,§^				
Higher	81.0	(444)	97.9	(83)
Secondary	61.3	(1061)	94.1	(184)
Secondary incomplete	53.5	(430)	72.8	(73)
Primary	40.0	(1625)	84.8	(306)
Primary incomplete	26.7	(1167)	78.4	(215)
No education	17.5	(945)	75.1	(163)
Mother’s occupation*				
Professional	53.5	(1126)	89.9	(210)
Sales	44.4	(392)	73.7	(72)
Agriculture	37.0	(1354)	81.5	(233)
Domestic	39.7	(189)	89.2	(30)
Manual	44.2	(319)	90.0	(60)
Services	82.9	(90)	94.0	(15)
Not working	42.5	(2574)	85.2	(494)
Father’s occupation*^,§^				
Professional	56.0	(1546)	91.2	(299)
Sales	47.1	(397)	88.7	(71)
Agriculture	29.7	(1690)	78.9	(288)
Domestic	31.1	(145)	89.6	(33)
Manual	48.9	(1508)	84.8	(261)
Services	50.3	(98)	64.4	(17)
Not working	46.5	(29)	67.7	(6)
Insurance coverage				
Yes	86.7	(299)	93.1	(48)
No	41.6	(5753)	84.7	(1067)

*p-value < 0.01 for skilled birth attendance.

^§^ p-value < 0.01 for measles immunization.

**Table 2:** Regression coefficients (b), concentration index (C) and contribution of determinants to wealth-related inequality in skilled birth attendance and measles immunization, Kenya, DHS 2008/09

		**Skilled birth attendance (N = 3506)**			**Measles immunization (N = 892)**	
**Determinants**	**b**	**C**	**Overall C = 0.14% contribution**	**b**	**C**	**Overall C = 0.08% contribution**
**Wealth quintile (ref: 1)**			**39.49**			**60.20**
2	0.21	−0.38	−2.00	0.22	−0.37	−2.99
3	0.46	0.01	0.15	0.68	0.02	0.36
4	0.60*	0.39	5.58	1.25	0.38	15.95
5 (richest)	1.70*	0.79	35.77	1.43	0.78	46.88
**Skilled antenatal care visits (ref: No)**			**9.38**			**5.07**
1-3	1.49*	−0.11	−8.75	0.96	−0.10	−8.25
4+	2.09*	0.14	18.13	1.00	0.14	13.32
**Sex : male**	**-**	**-**	**-**	0.01	−0.06	**−0.07**
**Age**	**-**	**-**	**-**	0.15*	0.01	**2.99**
**Birth order**	−0.10	−0.12	**5.62**	−0.37*	−0.13	**32.96**
**Mother’s age <20**	0.02	0.03	**0.04**	−0.31	0.00	**0.05**
**Rural residence**	−0.06	−0.18	**1.08**	0.65	−0.20	**−18.97**
**Province (ref: Nairobi)**			**5.66**			**−2.50**
Central	−0.43	0.30	−1.60	0.92	0.35	4.32
Coast	−0.10	0.07	−0.07	2.22	0.04	1.58
Eastern	−0.03	−0.09	0.05	−0.11	−0.11	0.36
North Eastern	0.41	−0.62	−0.81	1.69	−0.57	−4.06
Nyanza	−0.29	−0.09	0.58	0.04	−0.03	−0.04
Rift Valley	−1.64*	−0.11	5.92	1.38	−0.07	−5.52
Western	−0.83	−0.13	1.59	−0.31	−0.12	0.87
**Ethnic group (ref: Kikuyu)**			**4.99**			**−5.74**
Kalenijn	−0.03	−0.32	0.18	0.50	−0.31	−4.60
Kamba	−1.80*	−0.04	0.88	1.71	0.00	−0.12
Kisii	−1.05	−0.02	0.22	1.41	−0.03	−0.47
Luhya	−1.48*	0.01	−0.20	1.05	0.03	1.00
Luo	−1.24*	0.02	−0.50	0.65	0.05	1.06
Masai	−0.27	−0.33	0.16	−1.05	−0.41	1.40
Meru/Embu	−0.10	0.12	−0.09	1.99	−0.02	−0.40
Mijikenda	−1.70*	−0.13	1.45	0.71	−0.21	−1.57
Taita	−1.78	0.47	−1.07	−1.03	0.46	−1.18
Other	−1.59*	−0.26	3.96	0.23	−0.28	−0.87
**Religion (ref : Protestant)**			**−0.55**			**−0.54**
Catholic	−0.27	0.05	−0.34	0.06	0.02	0.03
Muslim	0.49	−0.13	−0.65	0.49	−0.09	−0.66
Other	−0.27	−0.42	0.44	−0.03	−0.46	0.09
**Married**	0.10	0.00	**0.03**	−0.27	0.00	**−0.19**
**Mother’s education (ref : Higher)**			**20.48**			**6.54**
Secondary	−1.91*	0.44	−11.73	−0.39	0.50	−4.09
Secondary. incomplete	−2.19*	0.14	−3.48	−0.80	0.15	−1.60
Primary	−2.41*	0.06	−5.75	−0.96	0.05	−3.16
Primary incomplete	−2.82*	−0.19	22.33	−0.72	−0.19	9.00
No education	−2.92*	−0.47	19.11	−0.78	−0.42	6.39
**Father’s education (ref : Higher)**			**7.71**			**21.68**
Secondary	0.06	0.23	0.42	−0.95	0.36	−14.72
Secondary. incomplete	−0.24	0.11	−0.27	−1.30	0.00	0.05
Primary	−0.40	−0.04	0.66	−1.42	−0.06	4.98
Primary incomplete	−0.62	−0.24	4.02	−1.75	−0.24	18.85
No education	−0.53	−0.54	2.88	−1.55	−0.53	12.52
**Mother’s occupation (ref : Professional)**			**1.40**			**1.74**
Sales	−0.06	0.12	−0.07	−0.80	0.07	−0.68
Agriculture	−0.13	−0.20	0.95	−0.17	−0.22	1.88
Domestic	−0.47	0.16	−0.25	0.14	0.01	0.00
Manual	−0.05	0.04	−0.01	0.81	0.05	0.45
Services	0.88	0.41	0.69	0.36	0.40	0.41
Not working	−0.10	−0.02	0.09	0.34	−0.01	−0.32
**Father’s occupation (ref : Professional)**			**1.21**			**2.22**
Sales	0.48	0.01	0.06	−0.50	0.04	−0.30
Agriculture	0.02	−0.29	−0.18	−0.40	−0.34	7.49
Domestic	−0.32	−0.09	0.10	−0.10	−0.05	0.03
Manual	0.38	0.10	1.33	−0.81	0.11	−4.66
Services	0.73	−0.02	−0.03	−2.07	0.27	−0.83
Not working	0.99	−0.33	−0.07	−4.58	−0.55	0.49
**Insurance coverage**	0.79	0.59	**3.47**	−1.05	0.58	**−5.44**

*p-value < 0.01. Regression coefficients were computed using a multivariate logistic regression model.

**Figure 3:**
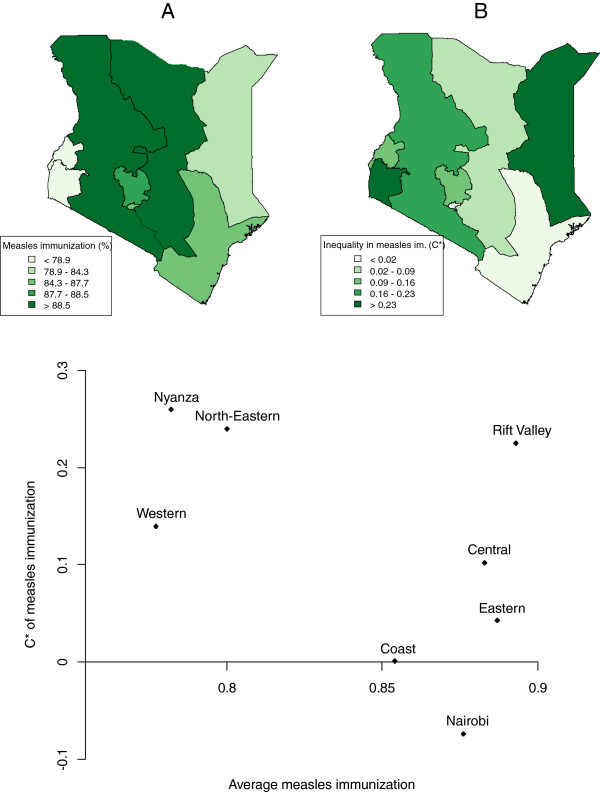
**Regional distributions of average (A) and inequality in (B) measles immunization, Kenya, DHS 2008/09.** * = Erreygers’ corrected concentration index [24].

Rift Valley and Eastern provinces had the highest percentages of measles immunization coverage, and Western and Nyanza, the lowest (Table 1, Figure 3A). Nyanza, North Eastern and Rift Valley had the highest wealth-related inequality in measles immunization (C* > 0.2) (Figure 3B). Nairobi was the only province with a negative C, i.e. an inequality favouring the poor. Coast, Central and Eastern had a high coverage (>85%) and a low inequality (C* < 0.11).

### Trends in wealth-related inequality in SBA and measles immunization

**Figure 4:**
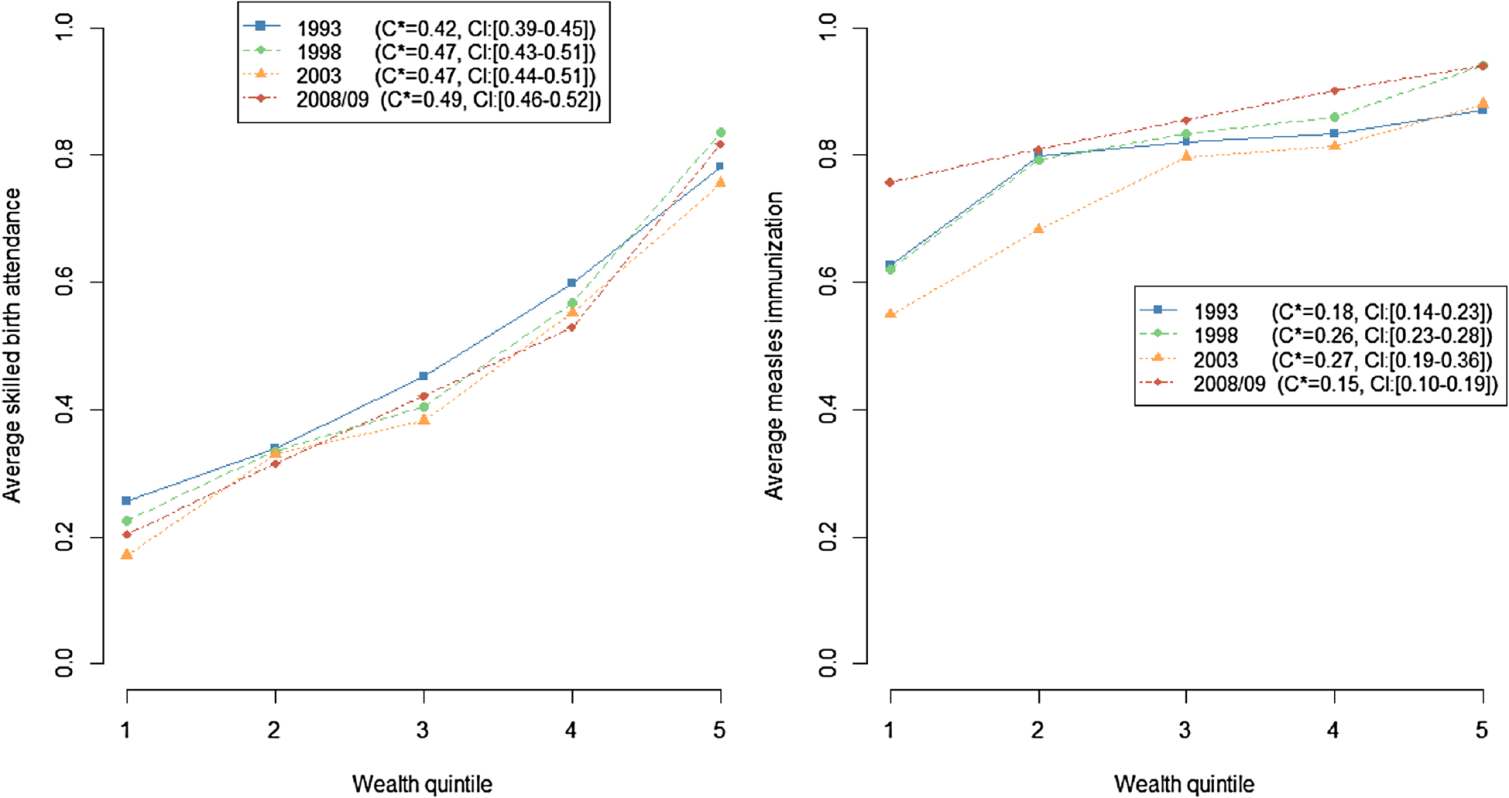
**Trends in wealth-related inequality in skilled birth attendance and measles immunization from 1993 to 2008/09, Kenya, DHS 1993, 1998, 2003, 2008/09.** * = Erreygers’ corrected concentration index [24].

Health care indicators were plotted as a function of wealth quintiles and a normalized relative C was computed, by survey year, in Figure 4. The wealth-related inequality in SBA was high in 1993 and persisted until 2008/09. SBA inequality showed a “mass deprivation pattern”, i.e., the majority of the population had equivalent but deficient access to SBA while small privileged groups (quintiles 4 and 5) had better access to this service. The pattern and the relative C varied little over the survey years.

The wealth-related inequality in measles immunization increased until 2003 and then dropped in 2008/09. The pattern took a “queuing” form, i.e., the general access to measles immunization was better than for SBA, but middle and richer wealth quintiles groups benefited most, while for the poorer groups a large proportion was still not vaccinated. In 2008/09, the queuing pattern seems to have diminished.

### Determinants of SBA

The distribution of SBA across population sub-groups is shown in Table 1, and regression coefficients from the multivariate analysis are shown in Table 2. In total, 44% of births were attended by a health professional.

SBA utilization increased with the household wealth quintile and with parent’s education. SBA coverage was four times higher in the richest quintile than in the poorest. Less than 20% of women with no education (13% of women) delivered with skilled assistance. This figure doubled when the mother had attained primary level education and tripled when the mother had attained secondary level education. SBA utilization was two times lower in rural households; however, the effect of rural residence faded when controlling for the other variables. Women from the Kikuyu ethnic group had the highest proportion of SBA uptake. In the multivariate analysis, women from the Kamba, Luhya, Luo and Mijikenda ethnic communities were less likely than Kikuyu to use SBA. Regardless of the socio-economic position of the household, SBA utilization decreased with birth order (p = 0.014), and increased with the number of antenatal care visits. Compared with women who had not attended antenatal care, it was three times greater when women had attended at least one visit, and about five times greater when women had attended the recommended four visits.

### Determinants of measles immunization

The distribution of measles immunization across population sub-groups is shown in Table 1, and regression coefficients from the multivariate analysis are shown in Table 2. Eighty-five percent of 12–23 months children were vaccinated against measles. Inequalities were less noticeable than for SBA, but nevertheless existed. Measles immunization increased with the household wealth quintile and with parent’s education. Children from the richest household wealth quintile and from parents with a secondary or higher education level were close to the 95% target. Coverage was below 80% in the following categories: poorest wealth quintile, parents with no formal education, Luo or Masai communities, father not working or working in the services or agricultural sector, Western province, no skilled antenatal care, birth order 4 and above. However, with the exception of birth order, the effect of socio-economic determinants on measles immunization was not statistically significant in the multivariate analysis.

### Decomposition of wealth-related inequality in SBA and measles immunization

The relative contributions to the overall C are shown in Table 2. A determinant can contribute to wealth-related inequality in health care both through its association with the health care indicator (regression coefficient) and through its unequal distribution across wealth groups (C). C indicates how unequally the determinant is distributed over wealth: if C > 0, the determinant is more prevalent among the wealthier, and if C < 0, the determinant is more prevalent among the poorer.

In 2008/09, the overall C for predicted SBA was 0.14. The wealth quintile accounted for about 40%. Twenty% of this inequality was explained by mother’s education. Other important contributors were antenatal care (9%), father’s education (8%), province (6%), birth order (6%), and ethnic group (5%).

For predicted measles immunization, the overall C was 0.08. Wealth itself accounted for 60% of this inequality. Other important contributors were birth order (33%), father’s education (22%), mother’s education (6%), and antenatal care (5%). In this decomposition analysis, some determinants had a negative contribution, meaning that they reduced the wealth-related inequality. Rural residence (−19%) was positively associated with measles immunization being more prevalent among the poor (negative C), whereas insurance coverage (−5%) was negatively associated with measles immunization but being more prevalent among the rich (positive C). Parent’s occupation was not a major source of inequality as a whole; only agricultural employee contributed notably to inequality in measles immunization.

## Discussion

The study investigated the effects of regional and socio-economic determinants on average levels (i.e., a level analysis), and the contribution of these determinants to wealth-related inequality (i.e., a gap analysis) of two health care indicators – SBA and measles immunization – in Kenya.

The results indicated that measles immunization coverage was relatively high in Kenya compared to other sub-Saharan African countries. Wealth-related inequalities of measles immunization were relatively low in the country but still higher than in countries like Zambia. On the other hand the SBA levels were among the lower in relation to other sub-Saharan African countries and the wealth-related inequalities were among the highest, however, Kenya was comparable to countries like Zambia and Madagascar. In a recent study comparing maternal and child health interventions inequality (relative C) in 54 countries, Kenya was ranked 18th for SBA and 17th for measles immunization [26].

Within Kenya large differences in coverage were also noticed between provinces for both indicators investigated. The SBA coverage in Nairobi (90%) (followed by Central province) was higher than in other provinces and in Nairobi the socio-economic inequalities were relatively low. This was to be expected given that the health facilities are easily accessible, unlike in Rift Valley where access to health facilities is usually a problem. Nairobi and Central province also showed relatively high coverage immunization levels and low inequalities. This study confirms the results of 2002 SIA [7] which also indicated that Nairobi and Central province improved measles immunization coverage equity. In 2008/09, all provinces were below the 95% SIA target and Nyanza and Western province were still below the 80% “routine” target, with especially high socio-economic inequalities in Nyanza.

SBA coverage or inequality did not remarkably vary over time. A significant improvement in measles immunization equity was observed in 2008/09. Following a nation-wide outbreak in 2005, SIA, first conducted in 2002, were followed up in 2006 [27]. These interventions seemed to have reached the poorest quintiles which were particularly left behind in the preceding surveys.

The level analysis indicated that the following socio-economic variables were significantly associated with SBA: household wealth, mother’s education and ethnic group. It has been noted that poor women face various barriers in the utilization of maternal health services: high costs of health services, poor transportation, inadequate health facilities, poor health decision making, insecurity at night in slums, or cumbersome hospital procedures (e.g. required proof of antenatal care attendance) [2,28]. Education has been reported to be a major determinant of maternal health care utilization [29]. Indeed, education improves the ability to evaluate where and when to seek care, and to correctly interpret and assimilate health messages [30]. As reported in other studies [31,32], women from different ethnic groups were less likely to use SBA compared to the Kikuyu. This community was identified as the most consistent in terms of their reproductive ideals and behaviors (e.g. low desired number of children) [33]. In this study, antenatal care attendance showed an important association with SBA use. Antenatal care interventions are an opportunity to reach pregnant women with messages and interventions, leading to improved maternal and newborn health [34].

The stratified analysis identified the characteristics of subgroups close to the 95% immunization coverage target: richest quintile, parent’s secondary or higher education level, and the subgroups far away from this target (<80%): poorest quintile, Luo or Masai communities, parent’s with no formal education, father not working or working in the services or agricultural sector, Western province, no skilled antenatal care, birth order 4 and above. Household wealth, parent’s education and father’s occupation were found to be associated with immunization in other sub-Saharan African countries [35-38].

The study also investigated the effects of determinants on the wealth-related inequality of SBA and measles immunization, i.e., what makes that poor people have lower levels. The wealth-related inequalities in both health care indicators were, apart from the direct effect of wealth itself, mainly due to differences in parent’s education. Antenatal care attendance and birth order were also important contributors; in addition to their effect on SBA and measles immunization levels, they were unequally distributed across wealth groups. As reported in [15], poor women were more likely to have more children. This last observation has been studied in [39] and was characterised as an inequity in itself. Province and ethnic group contributed more to SBA use than to measles immunization. Interestingly, rural residence reduced inequality in measles immunization by 19%. This is explained by the fact that, though more prevalent among the poor, rural residence had a positive effect on measles immunization. This could be a result of the SIA efforts in reaching geographically disadvantaged households by implementing vaccination sites (schools, churches, mosques) in the remote and rural areas of the country [7].

### Limitations

DHS are internationally comparable surveys [40] but the comparison with other sub-Saharan countries included DHS from different years between 2007 and 2012, possibly skewing the observed differences. Results of the trends analysis should be interpreted carefully because the available information differed between 1993/1998 and 2003/2008. Since 1999, women were asked to provide information about pregnancies resulting in live births during the five years prior to the survey. Before, the reference period was three years; the number of children included was lower. The study is limited to the information collected through DHS; other factors could have played a role in the analyses. Assessing access (cost and distance) to health facilities might be especially interesting when analysing inequalities in SBA. About 20% of household heads were not the parents of the child; their occupation may have been useful as well.

### Policy implications

This study brought to light several considerations when planning interventions aimed at improving health care coverage and equity.

Firstly, the decomposition analysis provided several areas to be targeted in order to reduce inequality in health care. These were: poverty reduction, educational attainment (preferably secondary level and above), antenatal care attendance and parity. Successful interventions targeting the consumer costs for transport and obstetric care barrier (e.g. community loan funds), mother’s education (e.g. community educators), or family planning (e.g. community-based delivery of services) were documented [30].

Secondly, the “mass deprivation” and “queuing” patterns of SBA and measles immunization presented in the trends analysis suggest that a broad strengthening of the whole system, possibly combined with targeting, is required [41]. A program targeting the rural poor was already implemented for measles immunization through the SIA, and was initiated in two districts of Nyanza in 2001 through the Skilled Care Initiative (SCI). SCI consists in the decentralization of routine and emergency obstetric care.

Thirdly, the analysis by province highlighted inequalities between provinces and wealth-related inequality within provinces. A more in-depth analysis determining the location of the most vulnerable sub-groups within provinces would help in better reaching the whole population during interventions.

Finally, each intervention should be consistent with the socioeconomic and political context which plays a proximate role in the process to equity illustrated in the conceptual framework proposed by the Commission on Social Determinants of Health (CSDH) [42]. The Kenyan context seems prone to changes for more equity in health. In the last decade, many initiatives were launched by the Kenyan government to improve social conditions and health, and some had an explicit equity goal [15]. Observations resulting from the study at hand are especially in line with the National Population Policy for Sustainable Development goals: improvement of the standard of living; health through education on how to prevent illness and premature death among mothers and children; sustenance of the on-going demographic transition to further reduce fertility; and responsible parenthood. Moreover, Kenya, as a country partner of the CSDH, was involved in the “Country Work Stream” aiming at turning evidence on the social determinants of health and health equity into effective policies. Whereas the National Reproductive Health Policy that was launched in 2007 did not overtly address issues of social determinants of health, the National Reproductive Health Strategy of 2009–2015 alluded to these. It was noted that the goal of reducing health inequities can only be achieved effectively by involving the population in decisions, mobilization, devolving and allocation of resources. The community strategy was aimed at enhancing community access to health care so as to improve productivity, which in turn would lead to reduction in poverty, hunger, child and maternal deaths [43]. Similarly the Second National Health Sector Strategic Plan of Kenya Annual Operational Plan 6 of July 2010–June 2011 reiterated the need to address equity through the community strategy [44]. However the results of the above efforts are yet to be realized.

## Conclusion

The two indicators used, i.e., SBA and measles immunization, seem to reflect two different aspects of the health system. Inequalities remain especially in SBA, reflecting the need of structural changes. Measles immunization inequalities were lower than for SBA and mostly explained by wealth itself. Nevertheless, such inequalities need to be tackled in order to achieve the >95% coverage in all districts target. Finally, both health care indicators require a broad strengthening with a special focus on disadvantaged sub-groups. All these issues have been addressed in the National Reproductive Health Strategy of 2009 – 2015.

## Competing interests

The authors declare that they have no competing interests.

## Authors’ contributions

CVM, IO and NS designed the study. CVM and IO analysed the data. NS and HVO supervised data analyses and results reporting. AK and SNM contributed to the interpretation of results and policy implications. CS contributed to data processing and creation of maps. CVM, IO and NS wrote the paper and all authors reviewed the manuscript.
